# Music processing in behavioural variant frontotemporal dementia and Alzheimer’s disease: a functional MRI study

**DOI:** 10.1093/braincomms/fcag196

**Published:** 2026-06-01

**Authors:** Jochum J van ‘t Hooft, Wietske van der Zwaag, Alle Meije Wink, Frederik Barkhof, Rebecca S Schaefer, Jason D Warren, Yolande A L Pijnenburg, Betty M Tijms

**Affiliations:** Alzheimer Center Amsterdam, Neurology, Vrije Universiteit Amsterdam, Amsterdam UMC Location VUmc, 1081 HZ Amsterdam, The Netherlands; Amsterdam Neuroscience, Neurodegeneration, 1081 BT Amsterdam, The Netherlands; Spinoza Centre for Neuroimaging, Netherlands Academy for Arts and Sciences, 1105 BK Amsterdam, The Netherlands; Computational Cognitive Neuroscience and Neuroimaging, Netherlands Institute for Neuroscience, 1105 BA Amsterdam, The Netherlands; Department of Radiology and Nuclear Medicine, Amsterdam UMC, Vrije Universiteit, 1081 HV Amsterdam, the Netherlands; Department of Radiology and Nuclear Medicine, Amsterdam UMC, Vrije Universiteit, 1081 HV Amsterdam, the Netherlands; UCL Institutes of Neurology and Healthcare Engineering, University College London, London WC1E 6BT, United Kingdom; Health, Medical and Neuropsychology Unit, Institute of Psychology, Leiden University, 2300 RB Leiden, The Netherlands; Academy for Creative and Performing Arts, Leiden University, 2300 RA Leiden, The Netherlands; Dementia Research Centre, UCL Queen Square Institute of Neurology, University College London, London WC1N 1AR, United Kingdom; Alzheimer Center Amsterdam, Neurology, Vrije Universiteit Amsterdam, Amsterdam UMC Location VUmc, 1081 HZ Amsterdam, The Netherlands; Amsterdam Neuroscience, Neurodegeneration, 1081 BT Amsterdam, The Netherlands; Alzheimer Center Amsterdam, Neurology, Vrije Universiteit Amsterdam, Amsterdam UMC Location VUmc, 1081 HZ Amsterdam, The Netherlands; Amsterdam Neuroscience, Neurodegeneration, 1081 BT Amsterdam, The Netherlands

**Keywords:** music perception, musicality, salience network, musicophilia, supplementary motor area

## Abstract

Music engages brain regions involved in perceptual, socio-emotional and cognitive functions that may be relatively preserved in individuals with Alzheimer's disease but seem affected early in behavioural variant frontotemporal dementia. The effects of music in dementia are often assessed through observational studies, leaving the neurophysiological underpinnings of music processing in these dementia types unclear. Improved understanding of these mechanisms is relevant because the effectiveness of music therapy may depend on dementia types. In this study we investigated whether patients with behavioural variant frontotemporal dementia and Alzheimer’s disease differ in music processing compared with healthy controls. We studied 60 participants (*n* = 35 female; aged 52–81), including 13 patients with behavioural variant frontotemporal dementia, 22 patients Alzheimer's disease, and 25 healthy controls. We designed a novel functional MRI paradigm based on passive listening to self-selected favourite music and experimenter-selected unfamiliar musical pieces using a sparse-sampling design. Activation patterns of favourite music listening (favourite > silence), unfamiliar music listening (unfamiliar > silence), and favourite music more than unfamiliar music (favourite > unfamiliar) were determined for each participant. Next, we compared activation patterns across groups for each contrast. Finally, associations between activation patterns and disease severity were investigated in behavioural variant frontotemporal dementia and Alzheimer’s disease separately. The patient groups exhibited typical neuropsychological, socio-emotional and structural anatomical changes associated with Alzheimer’s disease and behavioural variant frontotemporal dementia. Patients with behavioural variant frontotemporal dementia showed overall less activation during favourite music listening compared with Alzheimer’s disease and healthy controls. When contrasting favourite and unfamiliar music, we found that patients with behavioural variant frontotemporal dementia showed reduced activation in the supplementary motor area, a region that has previously been implicated as an important region for semantic musical memory. Increased connectivity of the auditory cortices was observed in behavioural variant frontotemporal dementia compared with controls, potentially indicating network immaturity. Only patients with Alzheimer’s disease exhibited activation in the caudate nucleus during unfamiliar music, a region associated with musical reward processing. Disease severity in Alzheimer’s disease and behavioural variant frontotemporal dementia were associated with distinct patterns of functional activation. Our results confirm and expand the observation that music is processed differently in patients with behavioural variant frontotemporal dementia and Alzheimer's disease. The reduced activation in the supplementary motor area may explain altered music processing in behavioural variant frontotemporal dementia. These differences in music processing could have clinical implications in the selection of music therapy.

## Teaser

This study investigated music processing in dementia using functional MRI. van ‘t Hooft *et al*. report that favourite music is processed differently in behavioural variant frontotemporal dementia compared with Alzheimer’s disease.

## Graphical Abstract

**Graphical Abstract:**
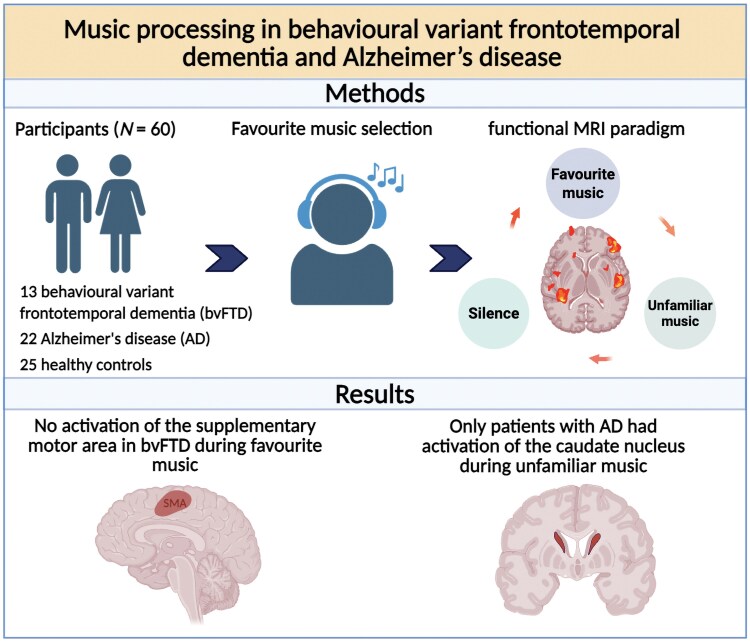

## Introduction

Behavioural variant frontotemporal dementia (bvFTD) and Alzheimer’s disease (Ad) are neurodegenerative disorders with distinct clinical syndromes and neuroanatomical profiles. Whereas bvFTD typically manifests through changes in personality and social conduct,^1^  Ad is typically characterized by changes in memory and general cognition.^2^ To address specific aspects of these disorders (e.g. mood complaints and agitation), large-scale music interventions are being implemented,^3^ which have demonstrated modest, but overall positive effects.^4^ Possibly, this reflects that aspects of music processing may be affected differently by certain dementia types. For example, musical semantic memory appears to be preserved in Ad^5,6^ but may be affected early in bvFTD. Improving understanding of these differences is relevant because the effectiveness of music-based therapies may depend on dementia types. However, the effects of music in dementia are often assessed through observational studies, and the neurophysiological underpinnings of music processing in these dementia types remain unclear.

Music processing is a multidimensional construct that can refer to perceptual, cognitive, and emotional processes that are engaged during music listening. Several aspects of underlying disease processes may impact music processing differences between Ad and bvFTD patients. For example, atrophy patterns that characterize bvFTD also include the core brain regions that are necessary for music processing in healthy participants, including frontoinsular, basal forebrain and mesolimbic structures.^7^ These brain regions are part of functionally defined large-scale networks, salience network (SN) and semantic appraisal network (SAN) that are targeted specifically by bvFTD.^8,9^ Damage to brain regions in these networks has been ascribed to impairments in music processing in bvFTD, such as music emotion recognition,^10,11^ rhythm and melody recognition,^11^ musical mentalizing,^12^ and recognition of musical deviants.^13^ In Ad on the other hand, these brain areas as well as the functional SN and SAN are relatively preserved.^9,14^ Moreover, in Ad noticeable changes in music processing have not been described. Rather, components of music perception seem to be relatively spared, such as musical semantic memory,^5,6^ and musical engagement,^15^ which may be a result of relative sparing of the salience network by Ad pathology.^5,16^ Based on these results it can be hypothesized that due to damage of the salience network, music processing is affected in bvFTD, and largely spared in Ad.

In the current study, we investigated music processing in bvFTD and Ad in relation to healthy controls using favourite music. Favourite music is commonly used in music-based interventions in dementia studies, for example when using personalised music playlists.^4,17^ Favourite music gives stronger and more robust physiological responses in healthy individuals,^18-20^ but whether these effects occur in bvFTD, and Ad remains unclear. Participants listened to self-selected favourite music as well as researcher-selected unfamiliar music using a novel fMRI paradigm. We hypothesized that favourite music processing is altered in bvFTD within regions of the salience network compared with Ad and controls, and that favourite music is relatively spared in Ad compared with controls.

## Materials and methods

### Participants

Participants from the Amsterdam Dementia Cohort (ADC) were included^21^ in a specialized tertiary cognitive clinic of the Alzheimer Centre Amsterdam. Patients with bvFTD and Ad were diagnosed in accordance with the current consensus criteria,^1,2^ with mild to moderate dementia severity. The control group consisted of participants with subjective cognitive decline (SCD), where no cognitive impairments were found on neuropsychological testing and amyloid biomarkers (CSF and/or PET) were negative. Subjects were excluded if they had subjective hearing problems, hearing aids, were unable to follow instructions during the MRI paradigm (e.g. due to cognitive impairments and/or language problems), or when they had neurodevelopmental, neurological or psychiatric comorbidity.

### Ethical approval and consent to participate

The ethics committees of the Amsterdam UMC location VUmc provided approval for the conduction of this work (submission 2022.0232) and all patients gave informed consent to participate in the research, and for the publication of their data. All participants signed informed consent in accordance with the declaration of Helsinki.

### Musical background, hedonic musical phenotypes, and hearing function

Musical background and musical sophistication (i.e. general musicality) were assessed using the Goldsmiths Musical Sophistication Index (GOLD-MSI) questionnaire.^22^ In line with previous studies,^11,23-25^ a questionnaire was used to assess musicophilia, which was defined as obsessive music listening, generally >10 h per week with a definite increase compared with premorbid levels. Informant-based questionnaires were used for the patient groups. After scanning all participants completed a short questionnaire detailing their experience (i.e. valence and arousal) when listening to their favourite music and judged the importance of music in their life on a 10-point scale. All participants underwent the digits-in-noise (DIN) hearing test that assessed speech in noise.^26^ The hearing test was performed with wired over-ear headphones (Devine Pro 4000) calibrated to 65 dBa.

### Cognitive and behavioural assessment

Participants underwent a standardized neuropsychological test battery. Memory was assessed with the Rey Auditory Verbal Learning Test immediate and delayed recall scores, and the Visual Association Test; Attention was assessed with the Trail Making Test A, and Stroop card 1 and 2; Language was assessed with Animal Fluency and the Visual Association Test naming condition; Executive functioning was assessed with the Trail Making Test B, Stroop card 3, and the Letter Fluency task. Domain *z*-scores were normalized to a reference group of 533 healthy amyloid-negative controls^27^ of the Amsterdam Dementia Cohort.^21^ Finally, informants completed the revised-self monitoring scale (RSMS), a measure for socio-emotional sensitivity.^28^

### Experimental stimuli selection and conditions

Participants selected a favourite music piece before the experiment. Favourite music was used because previous studies suggest that it is associated with robust, homogenous and personal functional response despite musical genre^18^ even in dementia.^17^ Ten seconds of the most recognizable parts of each music piece was selected by JH, generally the chorus. When in doubt, multiple selections were made, and participants could choose their favourite excerpt. The control conditions consisted of 10 s of unfamiliar music and 10 s of silence. The unfamiliar music consisted of an unknown royalty free music piece (https://www.bensound.com/royalty-free-music/track/acoustic-breeze) and was evaluated by 3 members of the research team for relative neutrality, with a low-arousal, positive mood, with few changes in dynamics or tonality. We had two versions to align with the selected favourite music piece to include vocals or not, to ensure this was always controlled for. Online software was used (www.bandlab.com) to adjust the loudness of the favourite music pieces to the same volume as the unfamiliar music. The selected favourite music pieces are displayed in Supplementary Table 1. During scanning, participants listened to their self-selected favourite musical pieces and unfamiliar musical pieces in a passive-listening fMRI design. Participants were instructed to listen to the music with their eyes open, focusing on a fixation cross. Stimuli were presented with a sampling rate of 44.1 kHz using in-house software running on MATLAB version: 9.13.0 (R2021a) (MathWorks, Inc., Natick, MA, United States). To reduce the effects of scanner noise, a sparse-sampling protocol with a long repetition time (TR) of 10 s was used. The complete stimulus set consisted of 24 blocks of favourite music, 24 blocks of unfamiliar music, and 24 blocks of silence, which were presented in a pseudorandomized order. The total functional MRI scanning protocol lasted 12:50 min.

### Brain image acquisition

Images were acquired on a 3T Philips Ingenia 3.0T CX whole body MRI scanner equipped with a 32-channel phased array coil. For each participant one run of 75 functional gradient-echo echoplanar image (GE-EPI) volumes were obtained using a sparse-sampling design. Each EPI volume contained 34 transversal images with a slice thickness of 2.7 mm, with an inter-slice gap of 0.27 mm, and a 2.7 × 2.7 mm in-plane resolution [echo Time (TE) = 30 msec, repetition time (TR) = 10 s, TRgap = 8 s], matrix slice 80 × 80 pixels, [field of view (FoV) = 216 × 216 × 101 mm]. For anatomical reference, structural volumetric T_1_-weighted images were obtained on the same 3.0T Philips scanner for each participant, with the following parameter set: 312 sagittal slices, TE = 4.48 msec, TR = 9.8 msec, inversion time (TI) = 950 msec, dimensions 256 × 240 × 250 mm, voxel size 0.8 × 0.8 × 0.8 mm.

## Data analyses

### Statistical analysis

Demographic data was analysed using R (version 4.1.0). Syndromic subgroup differences of demographic, music and hearing data were investigated using *t*-tests, and chi-squared tests used where appropriate. Subgroup differences of the neuropsychological and behavioural data were adjusted for age and sex. A criterion of *P* < 0.05 was used as a statistically significant difference in the group comparisons of demographic data.

### Voxel-based morphometry

The structural data of bvFTD patients and Ad patients were compared with the healthy controls using voxel-based morphometry (VBM) in order to investigate whether functional differences could be explained by atrophy patterns. Default parameter settings in SPM12 (https://www.fil.ion.ucl.ac.uk/spm/) were used for normalization, segmentation, modulation, and smoothing of grey and white matter images. A Gaussian smoothing kernel of 6 mm full-width-at-half-maximum (FWHM) was used for smoothing to match previous studies on music processing in dementia.^6,17,29^ Regional grey matter atrophy between the patient groups (bvFTD and Ad), and the healthy controls was investigated using a flexible factorial analysis of variance (ANOVA) with pairwise comparisons. Age, sex and total intracranial volume were included as covariates of no interest. Statistical parametric maps (SPMs) of grey matter atrophy were examined at a threshold of *P* < 0.05 after family-wise error (FWE) correction for multiple comparisons over the whole brain volume.

### Functional MRI data

The fMRI data was pre-processed and analysed using SPM12. The first dynamic volume was discarded to allow the fMRI signal to settle. Realignment (motion-correction) was done with the first image in the fMRI time series as reference. Subsequently, fMRI data were co-registered to the structural brain image and both structural and functional images were normalized to MNI standard stereotactic space and smoothed with a 6 mm FWHM Gaussian smoothing kernel. No slice timing correction or temporal filtering was performed due to the sparse-sampling design. Pre-processed images were entered into a first-level analysis, where the contrast images of favourite music (favourite > silence), unfamiliar music (unfamiliar > silence), and favourite versus unfamiliar music (favourite > unfamiliar) were calculated for each participant. First-level contrast images were entered into a second-level analysis to examine within- and between-group effects using a flexible factorial analysis of variance (ANOVA) with pairwise comparisons (bvFTD versus controls, Ad versus controls and bvFTD versus Ad). Age and sex were included as covariates of no interest. These analyses were repeated including additional covariates of no interest in cases where groups differed on these variables. A whole-brain threshold of *P* < 0.001 uncorrected for multiple comparisons with a minimum cluster size of 50 voxels was used. A post-hoc analysis within each patient group investigated whether disease severity was associated with activation patterns. We included measures for disease severity as a predictor and activation of favourite > unfamiliar music as outcome measure. In Ad we used the MMSE as a measure for disease severity, and in bvFTD the RSMS was used. A liberal threshold of *P* < 0.01 with a minimum cluster size of 30 voxels was used for this post-hoc analysis.

## Results

### Sample description

A total of 65 participants (14 bvFTD, 26 AD and 25 healthy controls) were recruited for this study. Two MRI scans were stopped prematurely due to anxiety and discomfort during scanning (Ad  *n* = 2), and three people (bvFTD *n* = 1, Ad  *n* = 2) were excluded due to motion artefacts. Table 1 describes the demographic and clinical data of all 60 included participants. Briefly, groups did not differ in average age, sex, handedness, musical training, musical sophistication, valence and arousal during their favourite music, and self-rated importance of music in their life. Patients with bvFTD and Ad had fewer education years (bvFTD < controls, *P* = 0.036; Ad < controls, *P* = 0.007), lower MMSE scores (Ad < controls, *P* < 0.0001; bvFTD < controls, *P* = 0.02) and lower RSMS scores (bvFTD < controls, *P* < 0.0001; Ad < controls, *P* = 0.003) compared with controls. Ad patients had lower MMSE scores compared with bvFTD (*P* < 0.001), and worse hearing function compared with controls (*P* = 0.0045), and bvFTD patients had lower RSMS scores compared with Ad (*P* = 0.006). The patient groups exhibited typical neuropsychological and socio-emotional profiles corresponding to either bvFTD or Ad. Seven bvFTD patients exhibited symptoms in line with musicophilia.

**Table 1:** Demographic characteristics

	HC	bvFTD	Ad	*P*-value
**Demographics**				
No. (M:F)	25 (7:18)	13 (8:5)	23 (11:12)	0.13
Age (years)	65.3 (5.8)	65.0 (5.7)	64.6 (7.1)	0.93
Education (years)	13.3 (2.5)	11.4 (2.9)	11.2 (2.3)	**bvFTD** **<** **Controls, *P*** **=** 0.036* bvFTD = Ad, *P* = 0.81 **Ad** **<** **Controls, *P*** **=** 0.007**
Handedness (R:L)	23:2	13:0	20:3	0.37
MMSE	29.5 (0.9)	27.0 (2.3)	22.0 (4.2)	**bvFTD** **<** **Controls, *P*** **=** 0.02^*^ bvFTD **>** **Ad, *P*** **<** 0.0001^***^ Ad **<** **Controls, *P*** **<** 0.0001***
Genetic mutations	n.a.	1 C9orf72, 3 MAPT, 1 SQSTM1, 1 TARDP	n.a.	n.a.
Symptom duration (years)	n.a.	4.4 (2.1)	4.9 (2.6)	0.69
**Hearing and music**				
Hearing function^a^	−8.0 (1.1)	−7.0 (2.0)	−6.5 (2.1)	bvFTD = Controls, *P* = 0.1 bvFTD = Ad, *P* = 0.4 **Ad** **>** **Controls, *P*** **=** 0.0045**
Musical training (7–49)^b^	16.5 (8.2)	15.1 (8.7)	15.1 (9.01)	0.78
Musical sophistication (18–126)^b^	61.1 (19.7)	57.1 (18.6)	56.3 (16.7)	0.77
Valence (1–10)	8.5 (1.4)	7.9 (2.6)	8.4 (2.23)	0.72
Arousal (1–10)	6.2 (3.0)	6.9 (2.5)	6.6 (2.41)	0.61
Importance of music (1–10)	7.65 (1.60)	8.2 (2.8)	7.7 (2.8)	0.57
Musicophilia (%)	0 (0%)	7 (58.3%)	0 (0%)	n.a.
**Socio-emotional and neuropsychological assessments**				
RSMS	45.1 (7.8)	25.1 (9.6)	35.6 (12.5)	**bvFTD** **<** **Controls, *P*** **<** 0.0001*** bvFTD **<** **Ad, *P*** **=** 0.0059*** Ad **<** **Controls, *P*** **=** 0.0033***
Attention domain *z*-score	0.64 (0.51)	−0.73 (0.77)	−1.41 (1.16)	**bvFTD** **<** **Controls *P*** **=** 0.0002*** bvFTD **=** **Ad, *P*** **=** **0.058** **Ad** **<** **Controls, *P*** **<** 0.0001***
Memory domain *z*-score	0.44 (0.78)	−1.2 (1.03)	−4.20 (2.06)	**bvFTD** **<** **Controls, *P*** **=** 0.0005*** bvFTD **>** **Ad, *P*** **<** 0.0001*** Ad **<** **Controls, *P*** **<** 0.0001***
Executive domain *z*-score	0.77 (0.83)	−0.86 (0.82)	−0.95 (0.92)	**bvFTD** **<** **Controls, *P*** **=** 0.0001*** bvFTD = Ad, *P* = 0.84 **Ad** **<** **Controls, *P*** **<** 0.0001***
Language domain *z*-score	0.43 (0.57)	−0.68 (0.37)	−0.70 (0.71)	**bvFTD** **<** **Controls, *P*** **=** 0.0002*** bvFTD = Ad, *P* = 0.97 **Ad** **<** **Controls, *P*** **<** 0.0001***

Data are presented as *N*, mean (SD). *Z*-scores of each cognitive domain were calculated in reference to a cognitively normal amyloid-negative control group (*n* = 533) (Groot *et al.*^27^). The RSMS and neuropsychological assessments were adjusted for age and sex.

Ad, Alzheimer’s disease; bvFTD, behavioural variant frontotemporal dementia; C9orf72, mutation in open reading frame 72 on chromosome 9; HC, healthy controls; MAPT, mutation in microtubule associated protein tau gene; MMSE, mini mental state examination; n.a., not applicable; n.s., not significant; RSMS, Revised Self-Monitoring Scale; SQSTM1, mutation in the sequestosome 1 gene; TARDBP, mutation within the 43-kDa transactive response (TAR)-DNA-binding protein.

^a^Hearing function was assessed with the digits-in-noise test.^26^ The Speech Reception Threshold (SRT) is shown in dB, a lower score depicts better hearing performance.

^b^The musical training score and the musical sophistication index of the GOLD-MSI were used; significant group differences are displayed in bold.

^*^ *P* ≤ 0.05.

^**^ *P* ≤ 0.01.

^***^ *P* ≤ 0.001.

### Structural neuroanatomical data

**Figure 1:**
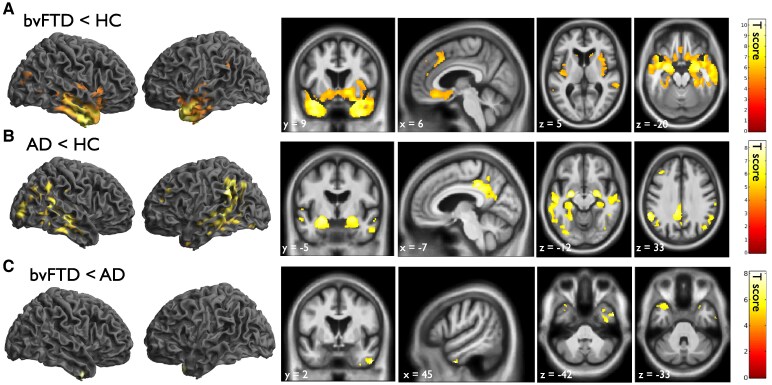
**Regional grey matter atrophy profiles of the bvFTD and Ad group.** (**A**) Grey matter atrophy of bvFTD patients compared with the healthy control group (bvFTD < HC). (**B**) Grey matter atrophy of Ad patients compared with the healthy control group (Ad < HC). (**C**) Grey matter atrophy of bvFTD compared with Ad (bvFTD < Ad). A flexible factorial analysis of variance (ANOVA) across the whole cohort (*N* = 13 bvFTD patients, 23 Ad patients and 25 healthy controls) with pairwise comparisons was used. SPMs are thresholded at *P* < 0.05 (after family-wise-error correction for multiple comparisons over the whole brain volume) and displayed on sections of the mean T1-weighted MR brain template image in MNI standard space; the left hemisphere is shown on the left of the axial and coronal sections. Ad, Alzheimer’s disease; bvFTD, behavioural variant frontotemporal dementia; HC, healthy controls.

The atrophy profiles of the bvFTD and Ad groups compared with the healthy control group are displayed in Fig. 1 and the associated MNI-coordinates can be found in the supplementary material (Supplementary Table 2). The bvFTD group had cortical atrophy in the temporal poles, the orbitofrontal cortex, basal forebrain, insulae, supplementary motor and ventromedial frontal regions. The Ad group had atrophy in mesio-temporal, parietal and occipital brain regions. When directly comparing bvFTD with Ad, we observed more atrophy in bvFTD in bilateral anterior temporal regions and the putamen (Fig. 1, Supplementary Table 2), these results are in line with expected atrophy patterns in Ad and bvFTD.

### Functional activation data

**Figure 2:**
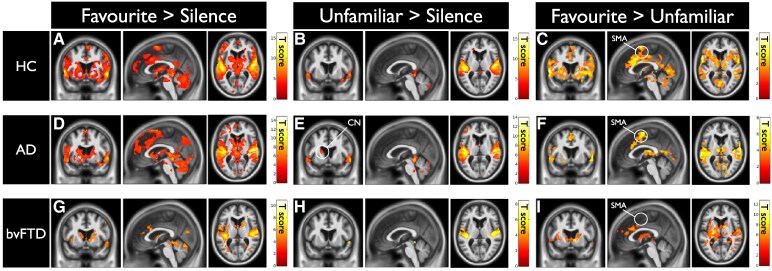
**Activation patterns of music in the diagnostic subgroups.** (**A-C**) Favourite, unfamiliar and favourite > unfamiliar in healthy controls. (**D-F**) Favourite, unfamiliar and favourite > unfamiliar music in Ad. (**G-I**) Favourite, unfamiliar and favourite > unfamiliar music in bvFTD. In each diagnostic subgroup (*N* = 13 bvFTD patients, 23 Ad patients and 25 healthy controls), the contrasts for favourite music (favourite > silence), unfamiliar music (unfamiliar > silence), and favourite > unfamiliar music were investigated using *T*-tests. Notable differences are indicated with white circles. SPMs are thresholded at *P* < 0.001 uncorrected for multiple comparisons over the whole brain volume. All figures are displayed on sections of a group mean T_1_-weighted MR brain template image in MNI standard space coordinates *x* = 2, *y* = 8, *z* = 6. *T* scores are coded on the colour bar. Ad, Alzheimer’s disease; bvFTD, behavioural variant frontotemporal dementia; CN, caudate nucleus; HC, healthy controls; SMA, supplementary motor area.

The functional activation patterns during music listening in each group are displayed in Fig. 2 and Supplementary Table 3. First, we inspected the main effects of the different conditions. Favourite music > silence showed the most widespread activation in all groups. In Ad and controls favourite music > silence showed activation patterns in the auditory cortices, cerebellum, brain stem, caudate nucleus, the putamen, ventromedial prefrontal cortex, the anterior cingulate cortex, insula, inferior frontal gyrus, precentral gyrus and lingual gyrus. In bvFTD activation patterns were restricted to the auditory cortices, cerebellum, caudate nucleus, brain stem and anterior cingulate cortex and were overall less widespread compared with Ad and controls. Unfamiliar music > silence showed overall less activation than the favourite > silence condition, and was restricted to activation of the auditory cortices, cerebellum, and brain stem. Additionally, activation of the inferior frontal gyrus was seen in Ad and controls, and only in Ad activation of the caudate nucleus was observed. Directly comparing favourite > unfamiliar music, we observed activation of the auditory cortices, the cerebellum, thalamus, anterior cingulate gyrus and precentral gyrus in all groups. In bvFTD and healthy controls only, there was additional activation of the caudate nucleus, while patients with Ad and healthy controls, but not the bvFTD patients, showed activation of the supplementary motor area (SMA).

**Figure 3:**
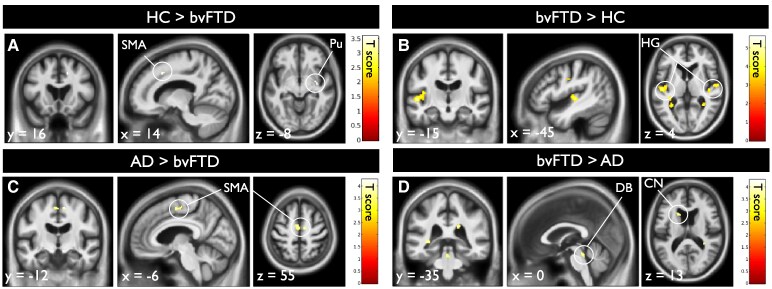
**Subgroup differences in favourite > unfamiliar music processing.** (**A**) More activation in the right SMA in healthy controls compared with bvFTD patients. (**B**) More activation in the auditory cortices in bvFTD patients compared with healthy controls. (**C**) More activation in the bilateral SMA in Ad patients compared with bvFTD patients. (**D**) More activation in the caudate nucleus and dorsal brain stem in bvFTD patients compared with Ad patients across the whole cohort (*N* = 13 bvFTD patients, 23 Ad patients and 25 healthy controls) with pairwise comparisons was used. SPMs are thresholded at *P* < 0.001 uncorrected for multiple comparisons over the whole brain volume and displayed on sections of a group mean T1-weighted MR brain template image in MNI standard space; the left hemisphere is shown on the left of the axial and coronal sections. *T* scores are coded on the colour bar. Ad, Alzheimer’s disease; bvFTD, behavioural variant frontotemporal dementia; CN, caudate nucleus; DB, dorsal brainstem; HC, healthy controls; HG, Heschl’s gyrus; Pu, Putamen; SMA, supplementary motor area.

Next, for each condition (i.e. favourite music > silence, unfamiliar music > silence and favourite music > unfamiliar music) we directly compared the activation patterns between the groups (Fig. 3 and Supplementary Table 4). Compared with controls, patients with Ad and bvFTD showed more activation in multiple temporal, frontal and parietal regions in both favourite music > silence and unfamiliar music > silence conditions, and more activation in the auditory cortex. Compared with bvFTD and HC, patients with Ad had more activation in the caudate nucleus in the unfamiliar music > silence condition and healthy controls showed more activation in the auditory cortex during the unfamiliar music > silence condition compared with bvFTD and Ad. Compared with Ad and controls, bvFTD patients had less activation in the SMA in the favourite music > unfamiliar music condition, and compared with Ad more activation in the dorsal brain stem, caudate nucleus and medial frontal gyrus. These results survived the analyses when adjusted for additional covariates (Supplementary Table 5).

**Figure 4:**
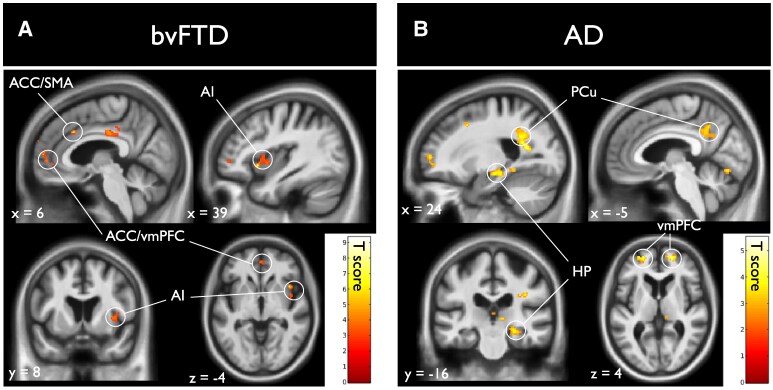
**Associations of favourite > unfamiliar activation patterns and disease severity.** (**A**) Association of favourite > unfamiliar activation patterns and RSMS total scores in bvFTD patients. (**B**) Associations of favourite > unfamiliar activation patterns and MMSE total scores in Ad patients. A multiple linear regression was performed, using RSMS scores to predict functional activation in bvFTD (*N* = 13) and MMSE scores to predict functional activation in Ad (*N* = 23). SPMs are thresholded at *P* < 0.01 uncorrected for multiple comparisons over the whole brain volume and displayed on sections of a group mean T1-weighted MR brain template image in MNI standard space; significant clusters >30 voxels are shown, the left hemisphere is shown on the left of the axial and coronal sections. ACC, anterior cingulate cortex; Ad, Alzheimer’s disease; AI; anterior insula; bvFTD, behavioural variant frontotemporal dementia; HP, hippocampus; MMSE, Mini Mental State Examination; PCu, precuneus; RSMS, Revised Self-Monitoring Scale; SMA, supplementary motor area; vmPFC, ventromedial prefrontal cortex.

Finally, we investigated whether activation patterns of favourite > unfamiliar music listening was related to disease severity as measured with the RSMS in bvFTD and MMSE in Ad. In patients with bvFTD, disease severity was associated with less activation in the right anterior insula, SMA, anterior cingulate cortex, and ventromedial prefrontal cortex (vmPFC). In Ad, disease severity was associated with less activation in the bilateral precuneus, the vmPFC, and right hippocampus (Fig. 4, Supplementary Table 6).

## Discussion

In the present study we tested the hypothesis that differences in music processing observed clinically in bvFTD and Alzheimer’s disease, may be associated with distinct patterns of brain activation on fMRI. The most widespread activation was observed during the favourite music condition. When testing group differences, the SMA was activated in controls and Alzheimer’s disease but not in bvFTD, and increased activation of the auditory cortices was found in bvFTD compared with healthy controls. The caudate nucleus and dorsal brainstem were activated in Alzheimer’s disease but not in bvFTD during unfamiliar music. Our results suggest distinct neural processing profiles for music in bvFTD and Ad, which may have implications for therapeutic applications.

In line with previous studies,^18-20,30-32^ we found that favourite music resulted in more activation than unfamiliar music. We extend these findings by demonstrating that this is maintained for Ad and bvFTD, albeit somewhat attenuated. Furthermore, activation patterns were related to disease severity. As suggested by previous studies,^10-13,23,24,33^ this suggests that music can be a useful paradigm for investigation of neurodegeneration. This is also demonstrated by the distinct patterns of activation that were related to disease severity in bvFTD and Ad. Whereas in bvFTD disease severity was associated with decreased activation in the right anterior insula and ACC, which are main hubs of the salience network, in Ad disease severity was associated with the precuneus, hippocampus and vmPFC, which are the main hubs of the default mode network. These networks are typically affected in bvFTD and Ad,^14,34^ and taken together, these results suggest that favourite music reactivity can index disease progression in bvFTD and Ad.

When comparing the activation patterns of favourite > unfamiliar music condition, we observed that individuals with bvFTD had less activation in the SMA compared with Ad and healthy controls. These results dovetail with previous findings that indicate that the SMA plays an important role for preserved semantic musical memory in Ad.^5,6,17^ Semantic musical memory has not been studied previously in relation to bvFTD, but the SMA was found to be involved in the impaired recognition acoustic and musical deviants in bvFTD patients.^13^ Furthermore, the SMA is closely connected to the salience network,^34,35^ which is typically affected in bvFTD.^9^ The favourite > unfamiliar music condition might partially represent musical familiarity of favourite music, which appears to be strongly salient and closely wedded to motor responses such as singing, moving and dancing along, especially in social settings.^5,6,36,37^ The SMA has been suggested to be a hub for multimodal integration of music,^36,38^ musical imagery,^39,40^ musical pleasure,^41^ emotional prosody,^36^ execution of musical behaviour^42^ as well as prosocial signals such as emotion recognition,^43,44^ and mirroring of contagious laughter.^37^ Although traditionally considered an area involved in motor functions, the involvement of the SMA across musical and social cognitive domains suggests it may support more general sequencing or integration processes, rather than solely reflecting covert motor activity. This fits well with the notion that music has a social function,^45,46^ and the SMA may play an important role in this. Finally, patients with bvFTD exhibited increased activation in the auditory cortices relative to healthy controls. These findings parallel observations in congenital amusia,^47-49^ a developmental disorder in musical pitch processing,^50^ and may indicate auditory network immaturity in bvFTD.^51^

The caudate nucleus and dorsal brainstem were activated in Ad during unfamiliar music, but not in bvFTD and healthy controls. This heightened activation during unfamiliar music may partially explain why Ad patients displayed a smaller difference in the favourite > unfamiliar music condition compared with bvFTD and healthy controls. This may also explain why activation in this region was observed in the favourite > unfamiliar contrast in bvFTD but not in Ad. The caudate nucleus is part of the musical reward system and is involved in anticipation of music.^32^ Additionally, we identified activation in the dorsal brainstem in the location of the locus coeruleus and raphe nuclei in Ad patients during unfamiliar music, which was not present in bvFTD and controls. One previous study found less activation in these nuclei in bvFTD compared with controls during musical mode variations.^29^ These nuclei are vital for coordinating arousing responses through afferent and efferent adrenergic and serotonergic pathways.^52^ Activation in the caudate nucleus and dorsal brain stem to unfamiliar music in Ad may suggest that patients with Ad may exhibit stronger emotional reactivity to any musical stimuli, including relatively neutral material. This has previously been suggested by a study that found stronger responses in the amygdala to neutral facial expressions.^53^ Additionally, these findings have potential implications for music therapy and other music-based interventions where favourite music is commonly used through personalized playlists.^4^ In all three groups favourite music had most widespread activation patterns, and personalized music playlists could form the basis for widely scalable music therapy interventions in dementia. However, the finding that unfamiliar and favourite music resulted in more robust activation in Ad compared with the other groups, might suggest that music-based interventions have more effects in Ad than bvFTD. Future studies should test this claim by investigating distinct responses to music-based interventions in bvFTD and Ad in order to tailor to the interventions to specific diseases.

A potential limitation of this study is the limited number of bvFTD patients that we were able to recruit during the study period, which may limit the statistical power to detect alterations in the bvFTD patients. Another limitation is the liberal thresholds that we used for our analyses, especially in the post-hoc analysis. Another potential limitation is the uncorrected threshold, as this can result in false positive results. While we did include only larger clusters of >50 voxels, follow up studies are needed to replicate results. There may also have been a selection bias for bvFTD patients that had more interest in music, which was demonstrated by 7 bvFTD patients that exhibited musicophilia. This may also have resulted in an underestimation of group differences if favourite music processing is relatively preserved in this group. Our control group consisted of patients with subjective cognitive complains, which may have influenced the results. The strength of this study was that we used personalized music, and that we used the same control conditions with unfamiliar music for all participants. Another strength was that we investigated the subjective experience (valence and arousal) of the favourite music. However, in future studies this could also be investigated in the control condition, as the contrast of favourite and unfamiliar music may be influenced by multiple components such as familiarity and saliency.

In conclusion, we found evidence that dementia subtypes have in distinct neural processing profiles for music, which may have implications for therapeutic applications. Future studies should investigate clinical implications of these differences in music processing.

## Supplementary Material

fcag196_Supplementary_Data

## Data Availability

Data and stimuli generated that support the findings of this study are available from the corresponding author, upon reasonable request. The MATLAB codes are available at https://github.com/Jochumvanthooft/HARMONIA_braincommunications.
