# Supplementary material for: Music processing in behavioural variant frontotemporal dementia and Alzheimer’s disease: a functional MRI study
Source: Brain Commun. 2026 Jun 1;8(3):fcag196. doi: 10.1093/braincomms/fcag196 (PMC13263052; doi:10.1093/braincomms/fcag196)
Supplement: fcag196_Supplementary_Data [file fcag196_supplementary_data.docx]

**Supplementary material**

**Music processing in behavioural variant frontotemporal dementia and Alzheimer’s disease: an fMRI study**

by J.J. van ‘t Hooft et al

**Supplementary Table 1. Favourite music pieces selected by participants**

| **Artist** | **Song** | **Lyrics** |
| --- | --- | --- |
| Direct | Soldier on | Yes |
| Deep Purple | Child in Time | Yes |
| Johann Sebastian Bach | Cello suite No 1. Prelude | No |
| Johann Sebastian Bach | Erbarme Dich (Matthäus Passion) | Yes |
| Claudia de Breij | Mag ik dan Bij jou | Yes |
| Benny Neijman | Vrijgezel | Yes |
| The Weeknd | Blinding Lights | Yes |
| Danny Vera | Rollercoaster | Yes |
| Rachmaninoff | Concert for Choir | Yes |
| Adele | Feel My Love | Yes |
| Paco Peña | Salobre | No |
| Kenny G | Forever in Love | No |
| Bee Gees | Too Much Heaven | Yes |
| Rene Karst | Supergave Tijd | Yes |
| Johan Strauss | An der Schönen Blauen Donau | No |
| Gluck | Orfeo ed Euridice | Yes |
| Blöf | Zoutelande | Yes |
| Frans Halsema | Voor Haar | Yes |
| André Hazes | Zeg Maar Niets Meer | Yes |
| Them | Here comes the night (ft. Van Morrison) | Yes |
| Frèdèric Chopin | Berceuse | No |
| Queen | Love Of My Life | Yes |
| Kenny Rogers | Lucille | Yes |
| Robbie Williams | Millenium | Yes |
| John Lennon | Imagine | Yes |
| Joe Jackson | Is She Really Going Out With Him | Yes |
| Bruce Springsteen | Spirit In The Night | Yes |
| Antonio Vivaldi | The four seasons (spring) | No |
| Queen | Bohemian Rhapsody | Yes |
| Santana | Maria Maria | Yes |
| Van Morisson | Moondance | Yes |
| Etta James | At Last | Yes |
| Frèdèric Chopin | Nocturne No. 3 Op. 6 | No |
| Véronique Sanson | Chanson sur ma drôle de vie | Yes |
| Tiels Vocaal Ensemble | Sure On This Shining Night | Yes |
| Giuseppe Verdi | Va Pensiero | Yes |
| Doris Day | Que Sera Sera | Yes |
| Nena | 99 Luftballons | Yes |
| Canyon | Mooi Volendam | Yes |
| Doris Day | Que Sera Sera | Yes |
| Rush | The Spirit Of Radio | Yes |
| Peer Gynt | Morning Mood | No |
| Wim Sonneveld | Aan De Amsterdamse Grachten | Yes |
| Simply Red | Sunrise | Yes |
| Eurythmics | Miracle of Love | Yes |
| The Brian Jonestown Massacre | Wisdom | Yes |
| Oscar Peterson | Blue Moon | No |
| Giuseppe Verdi | Va Pensiero | Yes |
| Phil Collins | In The Air Tonight | Yes |
| Frèdèric Chopin | March Funebre | No |
| Elvis Presley | In the Ghetto | Yes |
| Il Divo | Mama | Yes |
| Robbie Williams | Angels | Yes |
| Charles Aznavour | Il faut savoir | Yes |
| Sohne Mannheims | Und Wenn Ein Lied | Yes |
| Nightwish | 7 Days to The Wolves | Yes |
| Racoon | Liverpool Rain | Yes |
| Arik Lavi | Ma Kore | Yes |
| Alexander Papernyi | Tverboul | No |
| Candy Dulfer & David A. Steward | Lily was Here | No |

**Supplementary Table 2. Summary of regional grey matter atrophy in the patient groups**

|  | | | | **Coordinates** | | |  |
| --- | --- | --- | --- | --- | --- | --- | --- |
| **bvFTD < controls** | **Brain region** | **Side** | **Cluster (voxels)** | **x** | **y** | **z** | ***Z* score** |
|  | Temporal pole | R | 37722 | 52 | 2 | -40 | 7.73 |
|  | Amygdala | L |  | -30 | -3 | -24 | 7.56 |
|  | Anterior inferior temporal gyrus | R |  | 42 | -10 | -40 | 7.49 |
|  | Supplementary motor cortex | R | 525 | 3 | 22 | 40 | 5.91 |
|  | Supplementary motor cortex | R |  | 10 | 24 | 51 | 5.36 |
|  | Medial superior frontal gyrus | R |  | 8 | 30 | 34 | 5.11 |
|  | Angular gyrus | L | 166 | -58 | -56 | 36 | 5.33 |
|  | Middle occipital gyrus | R | 130 | 46 | -76 | 22 | 5.27 |
|  | Middle occipital gyrus | R | 72 | 40 | -86 | 14 | 5.11 |
|  | Anterior insula | L | 65 | -38 | 2 | 4 | 5.08 |
|  | Orbitofrontal gyrus | L | 32 | -39 | 21 | -21 | 5.29 |
|  | Temporal pole | L |  | -46 | 22 | -15 | 5.12 |
|  | Middle temporal gyrus | R | 45 | 60 | -56 | -4 | 4.83 |
|  | Supplementary motor cortex | L | 24 | -2 | 16 | 52 | 5.37 |
|  | Supplementary motor cortex | R | 18 | 3 | 44 | 20 | 5.37 |
|  | Middle temporal gyrus | L | 15 | -58 | -46 | 3 | 4.7 |
|  | Middle temporal gyrus | L |  | -62 | -39 | 0 | 4.63 |
|  | Medial superior frontal gyrus | R | 12 | 4 | 45 | 42 | 4.77 |
|  | Inferior temporal gyrus | L | 11 | -57 | -36 | -21 | 4.74 |
|  | Fusiform gyrus | R | 9 | 45 | -45 | -18 | 4.66 |
|  | Supplementary motor cortex | L | 7 | -2 | 18 | 39 | 5.05 |
|  | Medial superior frontal gyrus | R | 6 | 3 | 36 | 45 | 4.7 |
|  | Inferior temporal gyrus | R | 6 | 56 | -50 | -16 | 4.66 |
|  | Medial superior frontal gyrus | R | 4 | 12 | 46 | 34 | 4.71 |
|  | Middle temporal gyrus | L | 3 | -62 | -50 | -8 | 4.69 |
|  | Medial superior frontal gyrus | R | 3 | 3 | 48 | 2 | 4.68 |
|  | Temporal pole | L | 1 | -32 | 21 | -27 | 4.66 |
|  | Orbitofrontal gyrus | L | 1 | -30 | 18 | -27 | 4.66 |
|  | Angular gyrus | R | 1 | 63 | -51 | 22 | 4.62 |
|  | Fusiform gyrus | L | 1 | -34 | -48 | -12 | 4.62 |
|  | Medial superior frontal gyrus | R | 1 | 3 | 36 | 28 | 4.61 |
|  | Middle cingulate gyrus | R | 1 | 2 | -6 | 46 | 4.61 |
|  | | | | **Coordinates** | | |  |
| **AD < controls** | **Brain region** | **Side** | **Cluster (voxels)** | **x** | **y** | **z** | ***Z* score** |
|  | Supramarginal gyrus | L | 6824 | -63 | -46 | 32 | 6.75 |
|  | Middle temporal gyrus | L |  | -62 | -40 | 2 | 6.49 |
|  | Middle occipital gyrus | L |  | -46 | -70 | 15 | 6.41 |
|  | Middle temporal gyrus | R | 5526 | 51 | -32 | -4 | 6.3 |
|  | Middle temporal gyrus | R |  | 60 | -42 | 8 | 6.25 |
|  | Superior temporal gyrus | R |  | 57 | -33 | 2 | 6.04 |
|  | Hippocampus | L | 3830 | -26 | -8 | -14 | 6.57 |
|  | Hippocampus | L |  | -21 | -34 | -6 | 6.1 |
|  | Entorhinal cortex | L |  | -30 | 0 | -24 | 5.99 |
|  | Hippocampus | R | 2721 | 28 | -9 | -14 | 6.28 |
|  | Entorhinal cortex | R |  | 28 | 6 | -24 | 5.46 |
|  | Hippocampus | R |  | 24 | -15 | -21 | 5.46 |
|  | Posterior cingulate cortex | L | 2132 | -9 | -36 | 40 | 6.51 |
|  | Posterior cingulate cortex | L |  | -6 | -42 | 34 | 6.05 |
|  | Precuneus | L |  | -12 | -50 | 36 | 5.85 |
|  | Inferior occipital gyrus | R | 599 | 36 | -88 | 6 | 6.2 |
|  | Middle occipital gyrus | R |  | 38 | -84 | 16 | 5.23 |
|  | Middle frontal gyrus | L | 441 | -34 | 36 | 21 | 5.62 |
|  | Middle occipital gyrus | R |  | -38 | 32 | 34 | 5.42 |
|  | Middle frontal gyrus | L |  | -22 | 39 | 24 | 5.21 |
|  | Occipital fusiform gyrus | L | 271 | -32 | -80 | -14 | 5.59 |
|  | Occipital fusiform gyrus | L |  | -39 | -69 | -12 | 5.28 |
|  | Inferior occipital gyrus | L |  | -40 | -76 | -8 | 5.1 |
|  | Thalamus | R | 197 | 3 | -14 | 8 | 5.06 |
|  | Thalamus | R | 135 | 20 | -22 | 14 | 5.39 |
|  | Inferior temporal gyrus | R | 135 | 51 | -48 | -14 | 5.02 |
|  | Posterior insula | L | 99 | -40 | -15 | 2 | 5.62 |
|  | Posterior insula | L |  | -42 | -8 | -6 | 4.9 |
|  | Precuneus | L | 57 | -8 | -52 | 51 | 5.1 |
|  | Temporal pole | L | 56 | -42 | 20 | -22 | 5.33 |
|  | Temporal pole | L | 48 | -38 | 6 | -34 | 4.91 |
|  | Supramarginal gyrus | R | 45 | 64 | -27 | 33 | 5.15 |
|  | Supramarginal gyrus | R | 41 | 50 | -38 | 45 | 5.28 |
|  | Medial precentral gyrus | R | 34 | 8 | -28 | 45 | 4.88 |
|  | Superior parietal gyrus | R | 27 | 26 | -57 | 51 | 4.8 |
|  | Inferior temporal gyrus | L | 26 | -52 | -22 | -26 | 5.2 |
|  | Superior parietal gyrus | R | 25 | 34 | -48 | 46 | 4.91 |
|  | Middle occipital gyrus | L | 15 | -28 | -86 | 8 | 5.04 |
|  | Inferior occipital gyrus | R | 15 | 40 | -72 | -14 | 4.86 |
|  | Inferior temporal gyrus | L | 13 | -46 | -9 | -40 | 4.75 |
|  | Planum polare | L | 12 | -56 | -3 | 2 | 4.71 |
|  | Middle frontal gyrus | L | 11 | -32 | 48 | 3 | 5 |
|  | Supramarginal gyrus | R | 11 | 63 | -18 | 34 | 4.76 |
|  | Precuneus | R | 7 | 12 | -57 | 33 | 4.93 |
|  | Occipital pole | L | 7 | -9 | -98 | 15 | 4.87 |
|  | Planum temporale | R | 6 | 60 | -26 | 18 | 4.82 |
|  | Middle frontal gyrus | L | 6 | -34 | 16 | 30 | 4.81 |
|  | Fusiform gyrus | R | 6 | 39 | -60 | -12 | 4.74 |
|  | Inferior frontal gyrus | L | 5 | -38 | 9 | 24 | 4.73 |
|  | Supplementary motor cortex | R | 5 | 3 | 22 | 46 | 4.71 |
|  | Posterior insula | R | 4 | 33 | -18 | 4 | 4.85 |
|  | Fusiform gyrus | L | 4 | -36 | -8 | -36 | 4.75 |
|  | Middle frontal gyrus | R | 4 | 38 | 40 | 22 | 4.67 |
|  | Fusiform gyrus | R | 3 | 40 | -39 | -16 | 4.71 |
|  | Caudate nucleus | R | 3 | 10 | 22 | -3 | 4.67 |
|  | Precuneus | R | 2 | 14 | -52 | 44 | 4.72 |
|  | Superior frontal gyrus | L | 2 | -20 | 54 | 15 | 4.71 |
|  | Superior frontal gyrus | R | 2 | 24 | 45 | 32 | 4.65 |
|  | Planum polare | L | 2 | -60 | -20 | 9 | 4.64 |
|  | Supplementary motor cortex | R | 1 | 3 | 22 | 40 | 4.67 |
|  | Middle occipital gyrus | L | 1 | -45 | -80 | 21 | 4.64 |
|  | Middle frontal gyrus | R | 1 | 24 | 34 | 30 | 4.63 |
|  | Occipital pole | R | 1 | 22 | -94 | 2 | 4.62 |
|  | Middle frontal gyrus | R | 1 | 26 | 22 | 38 | 4.62 |
|  | | | | **Coordinates** | | |  |
| **bvFTD < AD** | **Brain region** | **Side** | **Cluster (voxels)** | **x** | **y** | **z** | ***Z* score** |
|  | Inferior temporal gyrus | R | 446 | 54 | 2 | -42 | 6.56 |
|  | Inferior temporal gyrus | R |  | 40 | -12 | -44 | 5.43 |
|  | Temporal pole | R |  | 34 | 8 | -45 | 5.1 |
|  | Temporal pole | L | 594 | -36 | 16 | -32 | 5.38 |
|  | Temporal pole | L |  | -40 | 20 | -39 | 5.23 |
|  | Temporal pole | L |  | -51 | 16 | -32 | 4.92 |
|  | Putamen | R | 43 | 16 | 9 | -8 | 4.89 |
|  | Middle temporal gyrus | R | 10 | 60 | -6 | -33 | 4.83 |
|  | Temporal pole | R | 38 | 42 | 10 | -22 | 4.81 |
|  | Temporal pole | R | 9 | 44 | 16 | -40 | 4.76 |
|  | Temporal pole | R | 17 | 30 | 16 | -34 | 4.71 |
|  | Temporal pole | L | 9 | -40 | 9 | -40 | 4.68 |
|  | Fusiform gyrus | R | 1 | 27 | -3 | -42 | 4.61 |

The regions shown were all thresholded at *P* < 0.05 (FWE-corrected for multiple comparisons) over the whole brain volume. Peak (local maximum) coordinates are shown in Montreal Neurological Institute (MNI) standard stereotactic space.

**Supplementary Table 3. Music processing in each diagnosis**

| **Group** | **Brain region** | **Side** | **Cluster size** | **Coordinates** | | | ***T* score** |
| --- | --- | --- | --- | --- | --- | --- | --- |
|  |  |  |  | **x** | **y** | **z** |  |
| **Favourite music > Silence** | |  |  |  |  |  |  |
| **HC** | Auditory cortex | R | 36939 | 50 | -16 | 8 | 16.13 |
|  | Auditory cortex | R |  | 50 | 4 | -4 | 15.62 |
|  | Auditory cortex | R |  | 52 | -10 | 2 | 14.66 |
|  | Supplementary motor cortex | R | 431 | 4 | 4 | 68 | 7.59 |
|  | Supplementary motor cortex | L |  | -6 | 2 | 64 | 6.68 |
|  | Medial frontal gyrus | L |  | -4 | 10 | 72 | 4.22 |
|  | Supramarginal gyrus | R | 185 | 56 | -28 | 46 | 6.35 |
|  | Supramarginal gyrus | R |  | 56 | -36 | 52 | 4.85 |
|  | Supramarginal gyrus | R |  | 52 | -44 | 50 | 4.25 |
|  | Calcarine cortex | L | 144 | -10 | -94 | -8 | 5.35 |
|  | Occipital pole | L |  | -14 | -100 | 6 | 5.11 |
|  | Calcarine cortex | L |  | -8 | -94 | 0 | 4.62 |
|  | Ventromedial frontal gyrus | R | 395 | 2 | 56 | 4 | 5.13 |
|  | Ventromedial frontal gyrus | R |  | 4 | 60 | 20 | 5.1 |
|  | Ventromedial frontal gyrus | R |  | 4 | 64 | 12 | 5.05 |
|  | Anterior insula | R | 83 | 24 | 34 | -2 | 4.66 |
|  | Ventromedial frontal gyrus | R |  | 20 | 44 | 2 | 3.95 |
|  | Calcarine cortex | R | 83 | 22 | -76 | 2 | 4.49 |
|  | Calcarine cortex | R |  | 22 | -66 | 2 | 4.37 |
|  | Inferior occipital gyrus | R |  | 34 | -68 | 4 | 3.64 |
|  |  |  |  |  |  |  |  |
| **AD** | Auditory cortex | R | 38346 | 48 | -16 | 4 | 14.78 |
|  | Auditory cortex | R |  | 42 | -24 | 8 | 13.92 |
|  | Auditory cortex | L |  | -44 | -26 | 8 | 12.75 |
|  | Superior occipital gyrus | R | 99 | 26 | -70 | 24 | 4.93 |
|  | Superior occipital gyrus | R |  | 22 | -78 | 34 | 4.77 |
|  | Cuneus | R |  | 22 | -74 | 18 | 3.89 |
|  | Caudate nucleus | R | 57 | 18 | 28 | 0 | 4.54 |
|  | Caudate nucleus | R |  | 10 | 24 | -6 | 4.54 |
|  | Anterior insula | R |  | 24 | 34 | 0 | 4.27 |
|  |  |  |  |  |  |  |  |
| **bvFTD** | Auditory cortex | R | 2193 | 62 | -12 | 4 | 10.85 |
|  | Auditory cortex | R |  | 42 | -22 | 10 | 10.25 |
|  | Auditory cortex | R |  | 50 | -14 | 2 | 9.31 |
|  | Auditory cortex | L | 3115 | -38 | -30 | 8 | 9.78 |
|  | Auditory cortex | L |  | -44 | -20 | 2 | 8.63 |
|  | Auditory cortex | L |  | -46 | -44 | 20 | 8.51 |
|  | Middle temporal gyrus | R | 97 | 38 | -56 | 10 | 8.68 |
|  | Inferior occipital gyrus | R |  | 36 | -70 | 6 | 5.46 |
|  | Inferior occipital gyrus | R |  | 42 | -66 | 2 | 4.64 |
|  | Brainstem | L | 842 | -4 | -34 | -14 | 8.43 |
|  | Brainstem | R |  | 6 | -34 | -8 | 7.97 |
|  | Ventral diencephalon | R |  | 4 | -26 | -8 | 6.14 |
|  | Inferior frontal gyrus | L | 204 | -38 | 38 | -2 | 7.86 |
|  | Inferior frontal gyrus | L |  | -32 | 32 | -8 | 7.44 |
|  | Inferior frontal gyrus | L |  | -40 | 28 | -4 | 6.69 |
|  | Middle frontal gyrus | R | 78 | 38 | 28 | 16 | 7.79 |
|  | Inferior frontal gyrus | R |  | 46 | 30 | 12 | 6.24 |
|  | Middle frontal gyrus | R |  | 32 | 38 | 16 | 5.37 |
|  | Cerebellum | R | 2030 | 32 | -56 | -24 | 7.73 |
|  | Cerebellum | L |  | -40 | -66 | -26 | 6.89 |
|  | Cerebellum | L |  | -8 | -62 | -14 | 6.83 |
|  | Precentral gyrus | R | 156 | 54 | -6 | 36 | 6.9 |
|  | Precentral gyrus | R |  | 52 | 4 | 48 | 6.07 |
|  | Caudate nucleus | R | 726 | 22 | 12 | 10 | 6.59 |
|  | Caudate nucleus | R |  | 8 | 4 | 2 | 6.11 |
|  | Caudate nucleus | R |  | 20 | 0 | 18 | 5.57 |
|  | Precentral gyrus | L | 117 | -40 | -18 | 38 | 6.05 |
|  | Postcentral gyrus | L |  | -54 | -14 | 36 | 5.77 |
|  | Precentral gyrus | L |  | -48 | -14 | 42 | 4.25 |
|  | Middle cingulate gyrus | R | 239 | 4 | 20 | 26 | 5.82 |
|  | Middle cingulate gyrus | L |  | -6 | 18 | 36 | 5 |
|  | Anterior cingulate gyrus | L |  | -6 | 24 | 30 | 4.25 |
|  | Caudate nucleus | R | 71 | 22 | 30 | -4 | 5.57 |
|  | Anterior insula | R |  | 10 | 30 | 0 | 5.39 |
|  | Frontal operculum | R |  | 32 | 34 | 6 | 4.62 |
|  | Anterior cingulate gyrus | L | 56 | -4 | 44 | 10 | 5.46 |
|  | Anterior cingulate gyrus | R |  | 2 | 38 | 18 | 5.07 |
|  | Precuneus | R | 50 | 24 | -54 | 6 | 4.83 |
|  | Lingual gyrus | R |  | 22 | -46 | 4 | 4.22 |
|  |  |  |  |  |  |  |  |
| **Neutral music > silence** | |  |  |  |  |  |  |
| **HC** | Auditory cortex | R | 3976 | 48 | -14 | 4 | 16.31 |
|  | Auditory cortex | R |  | 44 | -22 | 6 | 13.83 |
|  | Auditory cortex | R |  | 38 | -26 | 14 | 11.92 |
|  | Auditory cortex | L | 3528 | -50 | -4 | 0 | 15.44 |
|  | Auditory cortex | L |  | -38 | -28 | 10 | 15.14 |
|  | Auditory cortex | L |  | -50 | -24 | 6 | 13.79 |
|  | Brainstem | L | 534 | -4 | -36 | -8 | 9.89 |
|  | Ventral diencephalon | R |  | 16 | -26 | -4 | 7.23 |
|  | Ventral diencephalon | L |  | -16 | -26 | -6 | 5.98 |
|  | Ventromedial frontal gyrus | L | 56 | -12 | 54 | 20 | 6.69 |
|  | Ventromedial frontal gyrus | L |  | -16 | 46 | 18 | 4.2 |
|  | Cerebellum | L | 956 | -42 | -72 | -38 | 6.24 |
|  | Cerebellum | R |  | 20 | -78 | -40 | 5.62 |
|  | Cerebellum | L |  | -32 | -78 | -40 | 5.52 |
|  | Ventral diencephalon | R | 70 | 18 | -6 | -12 | 5.36 |
|  | Orbitofrontal gyrus | L | 93 | -20 | 36 | 6 | 5.21 |
|  | Orbitofrontal gyrus | L |  | -20 | 32 | -8 | 5.06 |
|  | Orbitofrontal gyrus | L |  | -12 | 28 | -6 | 4.56 |
|  |  |  |  |  |  |  |  |
| **AD** | Auditory cortex | R | 3254 | 50 | -14 | 2 | 13.9 |
|  | Auditory cortex | R |  | 62 | -2 | -2 | 10.78 |
|  | Auditory cortex | R |  | 50 | -20 | 8 | 10.44 |
|  | Auditory cortex | L | 4014 | -40 | -28 | 10 | 13.08 |
|  | Auditory cortex | L |  | -46 | -16 | 4 | 12.11 |
|  | Auditory cortex | L |  | -60 | -22 | 10 | 10.49 |
|  | Brainstem | R | 706 | 6 | -34 | -10 | 10.07 |
|  | Brainstem | L |  | -6 | -34 | -10 | 10.05 |
|  | Ventral diencephalon | L |  | -16 | -24 | -8 | 8.16 |
|  | Cerebellum | L | 3531 | -32 | -42 | -36 | 8.18 |
|  | Brainstem | L |  | -6 | -36 | -38 | 7.67 |
|  | Cerebellum | L |  | -26 | -46 | -28 | 7.66 |
|  | Middle frontal gyrus | L | 88 | -46 | 24 | 26 | 7.06 |
|  | Inferior frontal gyrus | L |  | -54 | 26 | 16 | 4.54 |
|  | Planum polare | L | 71 | -40 | 0 | -18 | 5.6 |
|  | Caudate nucleus | L | 162 | -16 | 4 | 12 | 5.37 |
|  | Caudate nucleus | L |  | -22 | 12 | 16 | 5.02 |
|  | Thalamus | L |  | -4 | -2 | 0 | 4.8 |
|  | Superior parietal gyrus | R | 146 | 30 | -42 | 38 | 5.28 |
|  | Precuneus | R |  | 20 | -46 | 46 | 4.74 |
|  | Angular gyrus | R |  | 36 | -46 | 44 | 3.99 |
|  | Superior parietal gyrus | L | 100 | -34 | -44 | 48 | 5.22 |
|  | Angular gyrus | L |  | -38 | -52 | 48 | 3.81 |
|  | Angular gyrus | R | 64 | 46 | -64 | 40 | 4.58 |
|  | Middle occipital gyrus | R |  | 36 | -72 | 40 | 4.05 |
|  | Angular gyrus | R |  | 36 | -58 | 46 | 4 |
|  |  |  |  |  |  |  |  |
| **bvFTD** | Auditory cortex | L | 1084 | -40 | -24 | 4 | 8.42 |
|  | Auditory cortex | L |  | -46 | -18 | 0 | 7.68 |
|  | Auditory cortex | L |  | -48 | -4 | -6 | 7.45 |
|  | Auditory cortex | R | 1066 | 42 | -22 | 8 | 7.89 |
|  | Auditory cortex | R |  | 48 | -14 | 4 | 7.78 |
|  | Auditory cortex | R |  | 50 | 0 | -4 | 7.3 |
|  | Cerebellum | R | 70 | 20 | -42 | -22 | 6.32 |
|  | Cerebellum | R |  | 30 | -44 | -28 | 4.36 |
|  | Brainstem | R | 60 | 6 | -34 | -8 | 5.51 |
|  | Brainstem | L |  | -4 | -36 | -8 | 5.26 |
|  |  |  |  |  |  |  |  |
| **Favourite music > Neutral music** | |  |  |  |  |  |  |
| **HC** | Precentral gyrus | L | 22097 | -56 | 6 | 6 | 8.76 |
|  | Precentral gyrus | R |  | 54 | -2 | 48 | 8.18 |
|  | Auditory cortex | R |  | 58 | -4 | -4 | 8.07 |
|  | Middle frontal gyrus | L | 363 | -30 | 32 | 26 | 6.29 |
|  | Middle frontal gyrus | L |  | -32 | 46 | 20 | 5.2 |
|  | Middle frontal gyrus | L |  | -38 | 32 | 30 | 4.83 |
|  | Superior parietal gyrus | R | 104 | 34 | -40 | 44 | 5.26 |
|  | Superior parietal gyrus | L | 459 | -34 | -42 | 44 | 5.22 |
|  | Superior parietal gyrus | L |  | -32 | -50 | 54 | 5.04 |
|  | Superior parietal gyrus | L |  | -26 | -58 | 50 | 4.97 |
|  | Precuneus | L | 172 | -8 | -66 | 32 | 5.16 |
|  | Superior occipital gyrus | L |  | -16 | -80 | 42 | 4.25 |
|  | Superior occipital gyrus | L |  | -18 | -82 | 34 | 4.08 |
|  | Precuneus | R | 107 | 20 | -72 | 38 | 4.81 |
|  | Superior parietal gyrus | R | 171 | 16 | -66 | 48 | 4.66 |
|  | Precuneus | R |  | 12 | -60 | 38 | 4.51 |
|  | Superior parietal gyrus | R |  | 22 | -60 | 36 | 3.97 |
|  | Middle occipital gyrus | L | 102 | -36 | -74 | 30 | 4.38 |
|  | Angular gyrus | L |  | -38 | -72 | 42 | 3.6 |
|  |  |  |  |  |  |  |  |
| **AD** | Auditory cortex | R | 5157 | 58 | -28 | 4 | 7.98 |
|  | Auditory cortex | R |  | 42 | -20 | 4 | 7.37 |
|  | Auditory cortex | R |  | 54 | -28 | 14 | 7.08 |
|  | Auditory cortex | L | 4577 | -40 | -20 | -2 | 7.84 |
|  | Auditory cortex | L |  | -56 | -10 | 0 | 7.46 |
|  | Auditory cortex | L |  | -38 | -34 | 10 | 7.32 |
|  | Supplementary motor cortex | R | 984 | 4 | -2 | 64 | 6.18 |
|  | Supplementary motor cortex | R |  | -1 | 4 | 56 | 6.1 |
|  | Supplementary motor cortex | L |  | -4 | -2 | 62 | 6 |
|  | Cerebellum | R | 871 | 12 | -64 | -22 | 5.93 |
|  | Cerebellum | R |  | 30 | -58 | -24 | 5.4 |
|  | Cerebellum | R |  | 20 | -66 | -18 | 5.4 |
|  | Cerebellum | L | 486 | -34 | -56 | -28 | 5.32 |
|  | Cerebellum | L |  | -22 | -66 | -20 | 5.1 |
|  | Cerebellum | L |  | -46 | -58 | -30 | 5 |
|  | Middle frontal gyrus | L | 116 | -24 | 44 | 20 | 5.02 |
|  | Middle frontal gyrus | L |  | -30 | 38 | 26 | 4.48 |
|  | Precuneus | L | 72 | -6 | -74 | 34 | 4.99 |
|  | Superior occipital gyrus | L |  | -14 | -78 | 38 | 3.74 |
|  | Inferior frontal gyrus | L | 54 | -44 | 8 | 24 | 3.91 |
|  | Middle frontal gyrus | L |  | -40 | 14 | 28 | 3.87 |
|  | Inferior frontal gyrus | L |  | -36 | 6 | 24 |  |
|  |  |  |  |  |  |  |  |
| **bvFTD** | Globus pallidus | R | 5531 | 12 | 4 | 2 | 12.72 |
|  | Thalamus | L |  | -26 | -18 | 8 | 11.23 |
|  | Anterior insula | L |  | -24 | 24 | -6 | 8.33 |
|  | Medial frontal gyrus | L | 875 | -6 | 26 | 42 | 9.48 |
|  | Anterior cingulate gyrus | R |  | 6 | 28 | 28 | 9.14 |
|  | Medial frontal gyrus | R |  | 1 | 44 | 20 | 7.64 |
|  | Auditory cortex | R | 1751 | 64 | -10 | 2 | 8.83 |
|  | Auditory cortex | R |  | 52 | -12 | 6 | 7.97 |
|  | Auditory cortex | R |  | 58 | -18 | 8 | 7.94 |
|  | Precuneus | L | 229 | -20 | -52 | 24 | 8.26 |
|  | Precuneus | L |  | -22 | -54 | 16 | 5.96 |
|  | Precuneus | L |  | -32 | -48 | 4 | 5.27 |
|  | Middle frontal gyrus | R | 148 | 34 | 30 | 18 | 7.44 |
|  | Middle frontal gyrus | R |  | 32 | 44 | 16 | 5.87 |
|  | Middle frontal gyrus | R |  | 24 | 46 | 8 | 5.24 |
|  | Inferior occipital gyrus | R | 58 | 36 | -70 | 6 | 6.54 |
|  | Middle occipital gyrus | R |  | 36 | -66 | 14 | 4.35 |
|  | Precentral gyrus | L | 161 | -38 | -16 | 38 | 6.22 |
|  | Precentral gyrus | L |  | -44 | -10 | 42 | 5.73 |
|  | Precentral gyrus | L |  | -46 | -8 | 30 | 4.77 |
|  | Superior frontal gyrus | R | 154 | 24 | 44 | 26 | 5.78 |
|  | Superior frontal gyrus | R |  | 26 | 52 | 22 | 4.97 |
|  | Superior frontal gyrus | R |  | 26 | 48 | 36 | 4.81 |
|  | Cerebellum | R | 190 | 32 | -60 | -22 | 5.77 |
|  | Cerebellum | R |  | 40 | -66 | -26 | 5.35 |
|  | Inferior frontal gyrus | R | 57 | 42 | 24 | -4 | 5.57 |
|  | Orbitofrontal gyrus | R |  | 36 | 24 | -10 | 4.2 |
|  | Ventral diencephalon | L | 55 | -10 | -20 | -14 | 5.4 |
|  | Ventral diencephalon | L |  | -20 | -16 | -10 | 5.36 |
|  | Cerebellum | L | 191 | -36 | -60 | -30 | 5.35 |
|  | Cerebellum | L |  | -28 | -60 | -24 | 5.1 |
|  | Cerebellum | L |  | -40 | -66 | -26 | 5 |
|  | Calcarine cortex | L | 64 | -22 | -70 | 6 | 5.05 |
|  | Calcarine cortex | L |  | -16 | -68 | 14 | 4.75 |

The regions shown were all thresholded at *P* < 0.001 (uncorrected for multiple comparisons) over the whole brain volume. All significant clusters >50 voxels are shown. Peak (local maximum) coordinates are shown in Montreal Neurological Institute (MNI) standard stereotactic space.

**Supplementary Table 4. Group differences in music processing**

| **Contrast** | **Group** | **Brain region** | **Side** | **Cluster size** | **Coordinates** | | | ***T* score** |
| --- | --- | --- | --- | --- | --- | --- | --- | --- |
|  |  |  |  |  | **x** | **y** | **z** |  |
| **Favourite music > silence** | **HC>bvFTD** | Anterior insula | R | 14 | 32 | 14 | -10 | 3.77 |
|  |  | White matter | R | 11 | 34 | -10 | -12 | 3.68 |
|  |  |  |  |  |  |  |  |  |
|  | **bvFTD>HC** | Posterior orbitofrontal gyrus | L | 72 | -26 | 30 | -6 | 5.84* |
|  |  | Calcarine cortex | L | 206 | -26 | -72 | 8 | 5.01 |
|  |  | Calcarine cortex | L |  | -22 | -82 | 8 | 3.98 |
|  |  | Inferior occipital gyrus | L |  | -42 | -72 | 6 | 3.66 |
|  |  | Corpus callosum | L | 30 | -2 | 24 | 0 | 4.85 |
|  |  | Corpus callosum | R |  | 8 | 28 | 0 | 4.5 |
|  |  | Temporal pole | L | 17 | -38 | 2 | -26 | 4.74 |
|  |  | Superior temporal gyrus | L | 36 | -56 | -6 | -4 | 4.48 |
|  |  | Supramarginal gyrus | L | 16 | -58 | -34 | 28 | 4.01 |
|  |  | Superior temporal gyrus | R | 15 | 48 | -6 | -14 | 3.87 |
|  |  | Posterior insula | L | 21 | -40 | -12 | 4 | 3.81 |
|  |  | White matter | R | 17 | 30 | -44 | 12 | 3.67 |
|  |  | Superior temporal gyrus | R | 10 | 58 | -4 | -4 | 3.66 |
|  |  | White matter/ventricle | L | 24 | -32 | -44 | 0 | 3.62 |
|  |  | White matter/ventricle | L |  | -32 | -52 | 6 | 3.48 |
|  |  | White matter/ventricle | L | 10 | -34 | -34 | 2 | 3.58 |
|  |  | Precentral gyrus | L | 16 | -48 | -8 | 36 | 3.57 |
|  |  |  |  |  |  |  |  |  |
|  | **HC>AD** | None |  |  |  |  |  |  |
|  |  |  |  |  |  |  |  |  |
|  | **AD>HC** | Orbitofrontal gyrus | L | 23 | -42 | 28 | -4 | 4.32 |
|  |  | Planum temporale | L | 37 | -44 | -34 | 4 | 4.15 |
|  |  | Middle temporal gyrus | L |  | -46 | -42 | 2 | 3.93 |
|  |  | Superior temporal gyrus | L |  | -50 | -38 | 8 | 3.39 |
|  |  | Inferior frontal gyrus | L | 35 | -52 | 26 | 16 | 4.02 |
|  |  | Cuneus | L | 16 | -6 | -80 | 36 | 3.93 |
|  |  | Postcentral gyrus | L | 20 | -58 | -12 | 34 | 3.82 |
|  |  | Precentral gyrus | R | 10 | 52 | -8 | 38 | 3.72 |
|  |  | Central operculum | L | 11 | -48 | 6 | 2 | 3.64 |
|  |  | Postcentral gyrus | R | 11 | 60 | -8 | 30 | 3.49 |
|  |  |  |  |  |  |  |  |  |
|  | **AD>bvFTD** | Angular gyrus | R | 12 | 40 | -66 | 44 | 3.63 |
|  |  |  |  |  |  |  |  |  |
|  | **bvFTD>AD** | Orbitofrontal gyrus | L | 24 | -24 | 28 | -8 | 4.58 |
|  |  | Middle cingulate gyrus | R | 49 | 8 | 10 | 26 | 4.52 |
|  |  | Calcarine cortex | L | 44 | -26 | -72 | 8 | 4.22 |
|  |  | Calcarine cortex | L |  | -22 | -82 | 8 | 3.61 |
|  |  | Thalamus | L | 10 | -6 | -14 | 0 | 3.79 |
|  |  | Middle cingulate gyrus | L | 11 | -4 | 16 | 28 | 3.7 |
|  |  |  |  |  |  |  |  |  |
| **Neutral**  **music > silence** | **HC>bvFTD** | Auditory cortex | R | 44 | 42 | -22 | 12 | 4.73 |
|  |  | Central operculum | R | 22 | 56 | -12 | 12 | 4.34 |
|  |  |  |  |  |  |  |  |  |
|  | **bvFTD>HC** | Temporal pole | L | 15 | -36 | 2 | -26 | 4.88 |
|  |  | Superior temporal gyrus | L | 33 | -56 | -6 | -4 | 4.16 |
|  |  | Fusiform gyrus | R | 62 | 24 | -40 | -18 | 3.88 |
|  |  | Cerebellum | R |  | 36 | -48 | -24 | 3.59 |
|  |  |  |  |  |  |  |  |  |
|  | **HC>AD** | Auditory cortex | R | 27 | 38 | -30 | 16 | 4.2 |
|  |  | Auditory cortex | R | 12 | 40 | -20 | 12 | 3.78 |
|  |  |  |  |  |  |  |  |  |
|  | **AD>HC** | Superior parietal lobule | L | 206 | -34 | -44 | 46 | 5.63* |
|  |  | Superior parietal lobule | L |  | -18 | -62 | 50 | 4.53 |
|  |  | Angular gyrus | L |  | -38 | -52 | 48 | 3.91 |
|  |  | Middle occipital gyrus | R | 61 | 36 | -72 | 38 | 5.37* |
|  |  | Middle occipital gyrus | R |  | 32 | -78 | 34 | 3.5 |
|  |  | Precuneus | R | 125 | 6 | -68 | 46 | 4.7 |
|  |  | Precuneus | L |  | -2 | -78 | 44 | 3.47 |
|  |  | Superior parietal lobule | R | 98 | 30 | -42 | 38 | 4.68 |
|  |  | Angular gyrus | R |  | 38 | -46 | 44 | 4.44 |
|  |  | Ventral diencephalon | L | 45 | -1 | -8 | -10 | 4.62 |
|  |  | Inferior frontal gyrus | L | 55 | -42 | 38 | -4 | 4.39 |
|  |  | Inferior frontal gyrus | L |  | -40 | 30 | 0 | 3.7 |
|  |  | Cerebellum | L | 35 | -26 | -44 | -28 | 4.34 |
|  |  | Precuneus | R | 133 | 18 | -74 | 38 | 4.29 |
|  |  | Superior parietal lobule | R |  | 22 | -62 | 42 | 4.22 |
|  |  | Angular gyrus | R |  | 30 | -66 | 46 | 3.43 |
|  |  | Inferior frontal gyrus | L | 50 | -44 | 18 | 8 | 4.22 |
|  |  | Supplementary motor gyrus | L | 28 | -4 | 12 | 58 | 4.2 |
|  |  | Superior temporal gyrus | L | 25 | -48 | -42 | 6 | 4.2 |
|  |  | Superior parietal lobule | L | 37 | -18 | -76 | 44 | 3.99 |
|  |  | White matter/ventricle | L | 10 | -30 | -36 | 6 | 3.87 |
|  |  | Postcentral gyrus | R | 77 | 30 | -28 | 40 | 3.83 |
|  |  | Middle frontal gyrus | L | 11 | -30 | 52 | 12 | 3.8 |
|  |  | White matter/ventricle | R | 36 | 14 | -28 | 32 | 3.59 |
|  |  | Posterior cingulate gyrus | R |  | 12 | -36 | 34 | 3.5 |
|  |  |  |  |  |  |  |  |  |
|  | **AD>bvFTD** | Angular gyrus | R | 13 | 42 | -52 | 44 | 3.9 |
|  |  | Frontal operculum | L | 18 | -44 | 18 | 10 | 3.87 |
|  |  | Superior temporal gyrus | L | 13 | -48 | -42 | 6 | 3.86 |
|  |  | Middle frontal gyrus | L | 22 | -42 | 44 | 6 | 3.79 |
|  |  | Middle frontal gyrus | L |  | -36 | 44 | 12 | 3.78 |
|  |  | Superior frontal gyurs | R | 10 | 14 | 52 | 34 | 3.55 |
|  |  |  |  |  |  |  |  |  |
|  | **bvFTD>AD** | Calcarine cortex | L | 10 | -4 | -78 | 6 | 3.83 |
|  |  |  |  |  |  |  |  |  |
| **Favourite music > neutral music** | **HC>bvFTD** | Supplementary motor cortex | R | 10 | 14 | 16 | 42 | 3.59 |
|  |  | Putamen | R | 12 | 32 | -4 | -8 | 3.58 |
|  |  |  |  |  |  |  |  |  |
|  | **bvFTD>HC** | Auditory cortex | L | 211 | -50 | -18 | 0 | 5.6* |
|  |  | Posterior insula | L |  | -40 | -16 | 12 | 4.26 |
|  |  | Auditory cortex | L |  | -58 | -6 | -2 | 3.49 |
|  |  | Orbitofrontal gyrus | L | 38 | -24 | 30 | -6 | 4.55 |
|  |  | Anterior insula | L | 32 | -40 | 16 | -12 | 4.37 |
|  |  | White matter/ventricle | R | 82 | 32 | -48 | 6 | 4.32 |
|  |  | White matter/ventricle | L | 46 | -34 | -34 | 2 | 4.31 |
|  |  | Calcarine cortex | L | 226 | -26 | -72 | 8 | 4.3 |
|  |  | White matter/ventricle | L |  | -30 | -50 | 6 | 4.08 |
|  |  | White matter/ventricle | L |  | -32 | -64 | 8 | 3.88 |
|  |  | Auditory cortex | R | 42 | 54 | -10 | 4 | 4.24 |
|  |  | Superior frontal gyrus | R | 16 | 20 | 60 | 14 | 4.21 |
|  |  | Thalamus | R | 81 | 2 | -6 | 18 | 4.12 |
|  |  | White matter/ventricle | R |  | 2 | 4 | 12 | 4 |
|  |  | White matter/ventricle | L | 21 | -12 | -6 | 22 | 4.11 |
|  |  | Auditory cortex | R | 18 | 42 | -20 | 6 | 3.97 |
|  |  | Caudate nucleus | L | 19 | -10 | 10 | 16 | 3.84 |
|  |  | Precentral gyrus | L | 14 | -54 | 0 | 18 | 3.69 |
|  |  | Precentral gyrus | L |  | -52 | 0 | 26 | 3.47 |
|  |  | Precentral gyrus | L | 17 | -48 | -8 | 34 | 3.62 |
|  |  |  |  |  |  |  |  |  |
|  | **HC>AD** | Posterior cingulate gyrus | R | 19 | 16 | -40 | 30 | 3.83 |
|  |  |  |  |  |  |  |  |  |
|  | **AD>HC** | Auditory cortex | R | 34 | 42 | -20 | 4 | 4.54 |
|  |  | Auditory cortex | L | 16 | -44 | -32 | 6 | 3.54 |
|  |  |  |  |  |  |  |  |  |
|  | **AD>bvFTD** | Supplementary motor cortex | R | 18 | 10 | -12 | 58 | 4.26 |
|  |  | Supplementary motor cortex | L | 46 | -4 | -6 | 56 | 3.87 |
|  |  | Supplementary motor cortex | L |  | -4 | -14 | 58 | 3.74 |
|  |  |  |  |  |  |  |  |  |
|  | **bvFTD>AD** | White matter/ventricle | R | 58 | 32 | -48 | 6 | 4.11 |
|  |  | White matter/ventricle | R |  | 30 | -40 | 20 | 3.59 |
|  |  | Caudate nucleus | L | 20 | -18 | 18 | 14 | 3.97 |
|  |  | White matter/ventricle | R | 41 | 18 | -38 | 26 | 3.96 |
|  |  | Superior frontal gyrus | R | 10 | 16 | 40 | 36 | 3.95 |
|  |  | White matter/ventricle | L | 22 | -34 | -36 | 2 | 3.89 |
|  |  | Dorsal brainstem | L | 28 | -2 | -34 | -20 | 3.8 |
|  |  | Anterior insula | L | 10 | -40 | 16 | -12 | 3.69 |

The regions shown were all thresholded at *P* < 0.001 (uncorrected for multiple comparisons) over the whole brain volume. * Significant at *P* 0.05 (FWE-corrected). The analyses were adjusted for age and sex. Peak (local maximum) coordinates are shown in Montreal Neurological Institute (MNI) standard stereotactic space.

**Supplementary Table 5. Group differences in favourite > neutral music processing with additional covariates**

| **Group** | **Region** | **Side** | **Cluster size** |  | **Coordinates** | | ***T* score** |
| --- | --- | --- | --- | --- | --- | --- | --- |
|  |  |  |  | **x** | **y** | **z** |  |
| **HC>FTD** | Putamen | R | 2 | 30 | -4 | -8 | 3.52 |
|  | Posterior insula | R | 2 | 36 | -10 | -8 | 3.52 |
|  |  |  |  |  |  |  |  |
| **FTD>HC** | Auditory cortex | L | 53 | -50 | -18 | 0 | 4.74 |
|  | White matter/ventricle | L | 62 | -32 | -64 | 8 | 3.95 |
|  | Calcarine cortex | L |  | -26 | -72 | 8 | 3.68 |
|  | Superior frontal gyrus | R | 8 | 20 | 62 | 14 | 3.86 |
|  | Fusiform gyrus | R | 2 | 42 | -6 | -26 | 3.84 |
|  | Anterior insula | L | 8 | -40 | 16 | -12 | 3.73 |
|  | White matter/ventricle | R | 17 | 34 | -48 | 6 | 3.7 |
|  | Posterior orbital gyrus | L | 4 | -24 | 30 | -6 | 3.68 |
|  | Hippocampus | L | 12 | -34 | -34 | 0 | 3.67 |
|  | White matter/ventricle | L | 25 | -32 | -52 | 6 | 3.64 |
|  | Auditory cortex | R | 4 | 58 | -30 | 14 | 3.58 |
|  | Posterior insula | L | 2 | -40 | -16 | 12 | 3.56 |
|  | White matter/ventricle | R | 12 | 2 | -6 | 18 | 3.51 |
|  | Supramarginal gyrus | L | 3 | -56 | -36 | 26 | 3.48 |
|  | Auditory cortex | R | 10 | 56 | -10 | 4 | 3.47 |
|  | Anterior insula | L | 2 | -34 | 8 | -10 | 3.45 |
|  | Orbitofrontal gyrus | L | 2 | -32 | 30 | -6 | 3.44 |
|  | Corpus callosum | L | 2 | -2 | 24 | 0 | 3.43 |
|  | Posterior insula | L | 1 | -42 | -12 | 4 | 3.34 |
|  | White matter/ventricle | L | 1 | -12 | -6 | 22 | 3.33 |
|  | Middle temporal gyrus | R | 2 | 44 | -12 | -18 | 3.31 |
|  | Ventricle | R | 5 | 4 | 4 | 12 | 3.29 |
|  |  |  |  |  |  |  |  |
| **AD>FTD** | Supplementary motor cortex | R | 17 | 10 | -10 | 58 | 4.19 |
|  | Supplementary motor cortex | L | 18 | -4 | -6 | 56 | 3.56 |
|  | Supplementary motor cortex | L |  | -4 | -14 | 58 | 3.45 |
|  |  |  |  |  |  |  |  |
| **FTD>AD** | White matter/ventricle | R | 24 | 32 | -48 | 6 | 3.91 |
|  | Caudate nucleus | L | 12 | -18 | 18 | 14 | 3.83 |
|  | White matter/ventricle | R | 26 | 18 | -38 | 26 | 3.82 |
|  | Medial frontal gyrus | L | 5 | -6 | 46 | 40 | 3.78 |
|  | Posterior orbital gyrus | L | 13 | -36 | 18 | -16 | 3.77 |
|  | Anterior insula | L | 3 | -24 | 28 | -6 | 3.7 |
|  | White matter/ventricle | L | 11 | -34 | -36 | 2 | 3.69 |
|  | Fusiform gyrus | R | 2 | 42 | -6 | -26 | 3.69 |
|  | Dorsal brain stem | L | 23 | -2 | -34 | -20 | 3.67 |
|  | Superior temporal gyrus | L | 6 | -50 | -20 | 0 | 3.6 |
|  | Calcarine cortex | L | 5 | -26 | -72 | 6 | 3.53 |
|  | White matter | R | 15 | 24 | 14 | 30 | 3.53 |
|  | White matter/ventricle | R | 19 | 30 | -42 | 20 | 3.52 |
|  | White matter/ventricle | R |  | 34 | -40 | 12 | 3.3 |
|  | Superior frontal gyrus | R | 3 | 16 | 40 | 38 | 3.48 |
|  | Globus pallidus | R | 4 | 12 | -2 | -4 | 3.45 |
|  | Calcarine cortex | L | 5 | -22 | -82 | 8 | 3.34 |
|  | White matter | R | 2 | 36 | -44 | 24 | 3.28 |
|  | Caudate nucleus | R | 1 | 14 | 16 | 16 | 3.27 |
|  |  |  |  |  |  |  |  |
| **AD>HC** | Supplementary motor cortex | R | 2 | 10 | -10 | 60 | 3.33 |
|  | Superior temporal gyrus | R | 1 | 58 | -28 | 12 | 3.28 |
|  |  |  |  |  |  |  |  |
| **HC>AD** | Posterior cingulate gyrus | R | 3 | 16 | -40 | 30 | 3.4 |
|  | Ventricle | L | 1 | -8 | 2 | 6 | 3.33 |
|  | Superior parietal lobule | L | 2 | -32 | -46 | 46 | 3.3 |

The regions shown were all thresholded at *P* < 0.001 (uncorrected for multiple comparisons) over the whole brain volume. The analyses were adjusted for age. sex. education years and hearing function. Peak (local maximum) coordinates are shown in Montreal Neurological Institute (MNI) standard stereotactic space.

**Supplementary Table 6. Associations of favourite > neutral music activation patterns and disease severity**

| **Group** | **Brain region** | **Side** | **Cluster size** | **Coordinates** | | | ***T* value** |
| --- | --- | --- | --- | --- | --- | --- | --- |
|  |  |  |  | **x** | **y** | **z** |  |
| **bvFTD** | Middle cingulate gyrus | R | 453 | 12 | -18 | 38 | 9.31 |
|  | Middle cingulate gyrus | L |  | -12 | -28 | 30 | 4.91 |
|  | Posterior cingulate gyrus | L |  | -12 | -38 | 28 | 3.61 |
|  | Supplementary motor area | R | 32 | 6 | 22 | 36 | 7.21 |
|  | Middle cingulate gyrus | L |  | -2 | 20 | 34 | 3.65 |
|  | Anterior insula | R | 92 | 40 | 16 | -4 | 7.07 |
|  | Anterior insula | R |  | 42 | 2 | -2 | 3.94 |
|  | Anterior insula | R |  | 36 | 6 | 2 | 3.93 |
|  | Medial frontal gyrus | L | 424 | -6 | 54 | 6 | 6.61 |
|  | Medial frontal gyrus | R |  | 18 | 60 | 12 | 6.27 |
|  | Anterior cingulate gyrus | L |  | 0 | 48 | 4 | 5.67 |
|  |  |  |  |  |  |  |  |
| **AD** | Ventral diencephalon | R | 67 | 10 | -8 | -20 | 5.5 |
|  | Superior frontal gyrus | L | 94 | -18 | 64 | 12 | 5.35 |
|  | Middle frontal gyrus | L |  | -28 | 58 | 4 | 4.08 |
|  | Middle frontal gyrus | L |  | -24 | 48 | 0 | 3.95 |
|  | Superior frontal gyrus | R | 187 | 18 | 56 | 6 | 5.27 |
|  | Frontal pole | R |  | 8 | 64 | 10 | 4.04 |
|  | Superior frontal gyrus | R |  | 22 | 56 | -2 | 3.83 |
|  | Middle frontal gyrus | R | 70 | 26 | 14 | 44 | 5.2 |
|  | Middle frontal gyrus | R |  | 36 | 24 | 46 | 3.5 |
|  | Superior parietal lobule | R | 1186 | 28 | -56 | 32 | 4.72 |
|  | Precuneus | R |  | 6 | -48 | 32 | 4.58 |
|  | White matter | R |  | 32 | -44 | 26 | 4.56 |
|  | Superior temporal gyrus | R | 48 | 48 | -4 | -20 | 4.52 |
|  | Middle temporal gyrus | R |  | 58 | 0 | -16 | 2.85 |
|  | Thalamus | R | 44 | 12 | -26 | 2 | 4.45 |
|  | Thalamus | R |  | 4 | -28 | 10 | 3.09 |
|  | Hippocampus | R | 109 | 24 | -20 | -12 | 3.96 |
|  | Ventral diencephalon | L | 31 | -12 | -10 | -22 | 3.65 |
|  | Lingual gyrus | L | 31 | -4 | -76 | -12 | 3.57 |
|  | Parietal operculum | R | 93 | 38 | -24 | 30 | 3.56 |
|  | Postcentral gyrus | R |  | 36 | -14 | 30 | 3.36 |
|  | Parietal operculum | R |  | 28 | -24 | 28 | 3.28 |
|  | Anterior cingulate gyrus | L | 62 | -14 | 40 | 18 | 3.55 |
|  | Anterior cingulate gyrus | L |  | -12 | 28 | 24 | 3.53 |
|  | Lingual gyrus | R | 38 | 22 | -40 | -8 | 3.34 |

The regions shown were all significantly associated with disease severity and thresholded all thresholded at *P* < 0.01 (uncorrected for multiple comparisons) over the whole brain volume. All significant clusters >30 voxels are shown. Peak (local maximum) coordinates are shown in Montreal Neurological Institute (MNI) standard stereotactic space.
